# Challenges in the Diagnosis and Management of Growth Hormone Deficiency in India

**DOI:** 10.1155/2016/2967578

**Published:** 2016-10-27

**Authors:** Mathew John, Ekaterina Koledova, Kanakatte Mylariah Prasanna Kumar, Harshal Chaudhari

**Affiliations:** ^1^Providence Endocrine and Diabetes Specialty Centre, Thiruvananthapuram, Kerala, India; ^2^Lead Endocrinology, Global Medical, Safety and CMO, Merck, Darmstadt, Germany; ^3^Centre for Diabetes and Endocrine Care, Bangalore, Karnataka, India; ^4^Biopharma, NDD and Endocrinology, Merck Specialties Pvt. Ltd., Mumbai, India

## Abstract

In clinical practice, every year approximately 150,000 children are referred with short stature (SS) based on a cut-off of fifth percentile. The most important endocrine and treatable cause of SS is growth hormone deficiency (GHD). The lack of reliable data on the prevalence of GHD in India limits estimation of the magnitude of this problem. The diagnosis and treatment of GHD are hurdled with various challenges, restricting the availability of growth hormone (GH) therapy to only a very limited segment of the children in India. This review will firstly summarize the gaps and challenges in diagnosis and treatment of GHD based on literature analysis. Subsequently, it presents suggestions from the members at advisory board meetings to overcome these challenges. The advisory board suggested that early initiation of the therapy could better the chances of achieving final adult height within the normal range for the population. Education and awareness about growth disorders among parents, regular training for physicians, and more emphasis on using the Indian growth charts for growth monitoring would help improve the diagnosis and treatment of children with GHD. Availability of an easy-to-use therapy delivery system could also be beneficial in improving adherence and achieving satisfactory outcomes.

## 1. Introduction

Growth hormone deficiency (GHD) is one of the most important endocrine and treatable causes of short stature (SS). Children with a height that is at least two standard deviations (SDs) below average or approximately the third percentile for that age and gender are deemed to have SS [1]. In the US alone, 90,000 children have height below the second percentile and are classified as having SS. Considering a cut-off of fifth percentile that is often used in clinical practice, about 150,000 children a year would be referred as having SS [2]. In a developing nation like India, perception of height as a marker of general health is less pronounced as compared to weight, due to the social health system being more focused on common causes of malnutrition, rather than on normal growth and development. This factor might lead to underdiagnosis of pathologic causes of SS and other conditions potentially associated with poor growth. Further, there is insufficient awareness of the need to measure height, height measurement techniques, and thresholds for referral among primary care physicians. It is often up to families to recognize that their child is not gaining proper height and seek advice from a primary care physician. In the majority of cases, the families wait until adolescence and late puberty, when chances to improve final height are limited.

The prevalence of GHD in children with SS ranges from 2.8% to 69% [3–6]. GHD is also estimated to be prevalent in more than 80% patients undergoing postneurosurgical procedure [7, 8]. In India, a study by Colaco et al. assessed the profile of GHD in 430 children in Bombay and found that 31% of the children had familial GHD and about 17% of children had idiopathic GHD [9]. These studies are biased by patterns of referral to tertiary centres of care. However, there is no pan-India study on the prevalence of GHD. The studies that have been conducted so far are few and regional [9, 10]. The lack of larger prevalence studies hinders accurate estimation of the magnitude of GHD problem. Knowing the prevalence would greatly help in streamlining the screening of children with GHD.

Diagnosis of GHD is based on a combination of auxology, biochemical analyses such as growth hormone (GH) stimulation tests and insulin-like growth factor 1 (IGF-1), skeletal age, magnetic resonance imaging (MRI), and exclusion of other systemic diseases which can have a similar presentation [16–18] (Table 1). Diagnosis of GHD is more challenging in resource constrained countries like India for various reasons. Recombinant human GH therapy was first approved for children with GHD in 1985 and later for the treatment of various conditions like idiopathic short stature (ISS), Turner syndrome (TS), Noonan syndrome (NS), Prader-Willi syndrome (PWS), chronic renal failure (CRF), and small for gestational age (SGA) [19–21].

In India, diagnosis and treatment of GHD are hurdled with various challenges restricting the availability of GH therapy to only a very limited segment of the children. In fact, the literature on GHD from India is also limited [22]. This review will look at the challenges in diagnosis and treatment of GHD in India. In March 2015, Merck Serono convened discussion forums in six Indian cities (Delhi, Bangalore, Kolkata, Hyderabad, Chennai, and Kochi) to identify the challenges in diagnosis and management of GH deficiency in the country. Leading experts in endocrinology discussed their views on overcoming these challenges. This article summarizes the salient discussion points of these meetings.

## 2. Methods

Challenges on diagnosis and management of the growth disorders were formulated based on a literature search conducted in databases including the US National Institutes of Health (PubMed), MEDLINE, Scopus, and Google Scholar and the findings were discussed at the forums of experts.

## 3. Results

The literature search identified gaps in diagnosis of GHD (suboptimal referral of children with growth disorders, poor recognition, and lack of proper training and education of physicians regarding growth disorders and lack of easy availability of latest diagnostic technologies with accuracy and reproducibility) and optimizing growth response and issues with patient adherence to therapy as the key challenges in the recognition and treatment of GHD.

### 3.1. Gaps in the Diagnosis of GHD

#### 3.1.1. Suboptimal Referral of Children with Growth Disorders

Suboptimal referral of children with growth disorders to the endocrinologist was agreed to be the single most important obstacle for diagnosis and management of GHD in India. Most pediatricians tend to focus on measuring weight rather than height despite the availability of Indian growth charts [31, 34] and guidance from the Indian Academy of Pediatrics (IAP). Advantages and disadvantages of Indian growth charts are presented in Table 2. For children under the age of 5 years, the Indian government and IAP have accepted the new standards for growth monitoring from World Health Organization (WHO) released in 2006 [35]. And, for the children in age group 5–18 years old, the revised IAP growth charts are recommended [31]. Several endocrinologists mentioned that insufficient medical and family history does not allow them to make accurate growth predictions. Others pointed out the Central Board of Secondary Education (CBSE) electronic health record data that has been collected but never used for referrals. It would be reasonable to select appropriate cut-offs (e.g., 3rd and 97th centiles) to get both short and tall children being referred to primary or secondary care with further investigations, for example, height SD score (HSDS) being calculated by nurses or pediatric doctors and a decision made for further referral to endocrinologists. In studies from India, the mean age of patients diagnosed with GHD for various trials range from 8.6 to 14 years (Table 1). However, in general clinical practice, outside of clinical trial settings, most physicians participating in the advisory boards were commenting on late referral for the first consultancy of children with growth disorders. To the best of our knowledge, no literature is available on late referral of children with growth disorders in India. However, it is acknowledged that patients with hypothyroidism have been referred late as well [36]. This suggests the need for a threshold for referral in order to identify growth abnormalities in children.

The SD score (SDS) used in the detection of SS is helpful in distinguishing between normal and SS due to GHD. The Dutch consensus guidelines (DCG) interpreted the cut-off value for referral as SDS <−1.3 SD in order to identify the risk groups that need further evaluation. In addition, the guidelines provide several other referral criteria including clinical symptoms, persistent SS after being born SGA, height standard deviation score (HSDS), and growth deflection [37, 38]. A study by Grote et al. compared the referral criteria of the DCG with those of the UK Consensus Guideline (UKCG) and the WHO Global Database on Child Growth and Malnutrition cut-off values. The study concluded that too many children aged <18 years (nearly 80%) would be referred if we use DCG, whereas use of the UKCG leads to only 0.3% referrals and the WHO criteria to approximately 10% [38].

**Figure 1:**
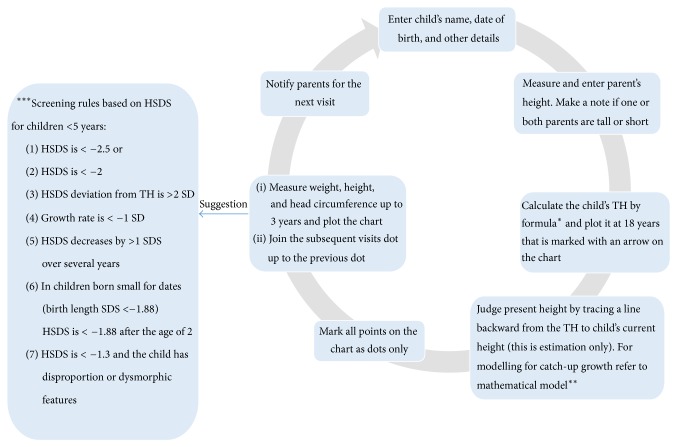
Guidelines for plotting growth charts as per guidelines from the Indian Academy of Pediatrics. ^*∗*^Tanner et al. [11]; ^*∗∗*^Boersma et al. [12]; ^*∗∗∗*^van Buuren et al. [13]; Saari [14]. TH: target height; HSDS: height standard deviation score; SD: standard deviation; SDS: standard deviation score.

**Figure 2:**
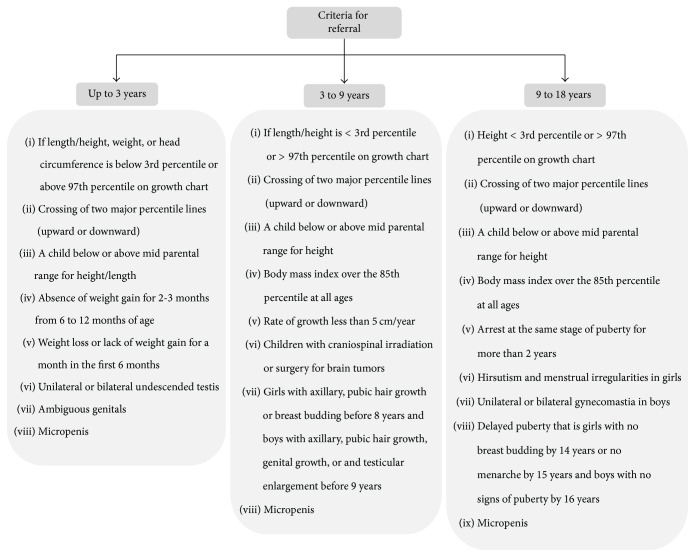
Criteria for referral.

In a consensus document by the IAP, clear guidelines are given on growth monitoring, plotting on growth charts, and criteria for referral (Figures 1 and 2). However, implementation of these guidelines is not satisfactory [34]. The WHO has also developed growth monitoring charts; and a training course is available to help in assessing child growth [39]. Haymond et al. suggested that, for a good differential diagnosis, it is essential to get medical history, family history, physical examination, analysis of the growth curve, and weight-for-height measurements [40]. A review by Nwosu and Lee on evaluation of short and tall stature in children suggested that firstly a thorough history and physical examination should be conducted and the laboratory investigations should then be based on the finding of these examinations [1].

In India, children are followed up for immunization and minor illness by general practitioners and pediatricians in most areas. Weight recording to identify protein-energy malnutrition (PEM) has been ingrained in pediatric practice, as PEM is still a major public health problem in India [41]. Height measurement is also critical to assess wasting (weight for height) and stunting (height for age) [41]. This is justified by the study of growth retardation by Nath et al. where PEM and chronic anemia resulted in more than 60% of cases of SS [42]. In various endocrine causes of growth failure like GHD including hypopituitarism, primary hypothyroidism, precocious puberty, and other rare congenital genetic disorders (e.g., NS, SS homeobox-containing gene deficiency (SHOX-D), and TS), there is a failure to gain height but the weight continues to be within normal centiles [43, 44].

#### 3.1.2. Poor Recognition of Growth Disorders by Pediatricians and Public

The other important factor that contributes to delay in diagnosis of growth disorders is poor recognition and understanding of growth disorders by pediatricians. This was closely associated with delayed societal alertness, whereby families only start to worry about the short height of their child at the late adolescent age. At this stage, the growth potential is greatly diminished and the efficacy of GH treatment interventions is limited [40].

#### 3.1.3. Current Technologies for Diagnosis of Growth Disorders

Although several diagnostic tools are available for the diagnosis of growth disorders, none of them can be completely relied upon to confirm the diagnosis [45–47]. Tests used for the diagnosis of GHD include auxology, measurement of IGF-1 and IGF binding protein 3 (IGFBP-3), radiographic assessment of bone age, cranial MRI, GH provocation testing, and genetic testing [45–47]. IGF-1 and IGFBP-3 are other commonly suggested tools for screening (or confirmation) of GHD. IGF-1 measurement is limited because of the sensitivity of the assay and the results may be inaccurate because IGF-1 circulates as a complex with acid labile subunit (ALS) or with IGFBP-3 or other IGFBPs. Although IGFBP-3 is inactivated or conventionally removed before assay, the removal is incomplete. Thus, IGFBP affects accuracy of both competitive and noncompetitive assays [45].

Juul and Skakkebaek conducted a study to assess the outcome of IGF-1 and IGFBP-3 in screening children for GHD. In children <10 years, the sensitivity of IGF-1 and IGFBP-3 was reported to be 53.3% and 60%, respectively. Both the tests had specificity of 97.9% [48]. Another study by Cianfarani et al. also showed that IGF-1 and IGFBP-3 possessed a sensitivity of 73% and 30%, respectively. The specificity for IGF-1 was 95% and for IGFBP-3 was 98% [49]. These studies have inferred that both these tests possess good specificity but lack sensitivity. However, in India there is a lack of availability of these assays in semiurban and rural areas. Poor standardization of these assays in commercial laboratories in India is yet another limitation. Although normative IGF-1 data for Indian children has been derived, it is seldom used in commercial laboratory reports [25].

GH provocation testing is one of the other methods of diagnosing GH deficiency. Despite their limitations in terms of types of provocation stimuli, the need for sex steroid priming, cut-off levels for GHD diagnosis, assay related problems, and lack of normative data, GH provocation testing is commonly used by most practitioners for decision-making on initiating treatment [50]. In India, clonidine is the most commonly used GH provocation stimulus. The insulin tolerance test is labour intensive and most advisors felt that it is impractical outside an academic setup. Rarely, glucagon is used as GH provocation stimulus. Other stimuli, such as GH releasing peptide-2 (GHRP-2), arginine, and L-dopa, are not available in India routinely. Cranial MRI is another significant tool for the diagnosis of GHD. However, physicians should not underestimate clinical clues that increase the likelihood of abnormal MRI findings and congenital pituitary hormone deficiencies, particularly with regard to facial dysmorphology and more common clinical syndromes [51]. Genetic testing might serve as an important diagnostic tool in the future. Outside India, GH1 and GH releasing hormone receptor (*GHRHR*) mutations have been identified in several clinical studies to be associated with GHD and familial cases of SS [45]. Genes, for example,* HESX1*,* PROP1*,* POU1F1*,* LHX4*, and* LHX3*, could also be considered based on the probability of identifying a mutational lesion that may be responsible for phenotype [52]. In India, Desai et al. in their study of 31 patients with GHD reported that 22 (71%) of the patients had a homozygous G to T transversion in exon 3 [53]. The majority of the patients (71%) had an* E72X *mutation in the* GHRHR *gene. Other genes known to be associated with SS include* PTPN11*,* SOS1 *(NS),* FGFR3 *(achondroplasia and hypochondroplasia),* SHOX* (SHOX-D),* NPR2*, aggrecan, and* PAPPA2* [54–56]. SHOX deficiency is the first indication being approved for GH treatment that requires genetic testing [20]. The availability of genetic testing for SS syndrome is limited outside metropolitan cities and is expensive.

The Growth Genetics Consortium (GGC), an international collaborative effort, has created a public database and website which includes information on molecular defects of the GH-insulin growth factor (GH-IGF) axis. This database can provide guidance to healthcare professionals for identification, evaluation, and management of patients with defects of the GH-IGF axis. Thus, GGC can be helpful in diagnosing the underlying genetic defects for SS [57].

### 3.2. Challenges in the Treatment of GHD

GH therapy is the mainstay treatment for growth disorders [58]. Somatotropin, a recombinant GH, is used for treatment of several conditions including GHD, TS, ISS, SGA, PWS, CRF, and NS. However, inappropriate growth response to GH treatment has been observed in clinical studies [59, 60]. Multiple factors result in an inappropriate response to GH therapy including significantly late initiation of the therapy and dosage limitations imposed by regulatory authorities. Variability in response to treatment from person to person may be due to several characteristics including diagnosis, body composition, age, and several other exogenous and endogenous factors [61].

Optimization of GH therapy is a prime challenge in the treatment of GHD. It requires evaluation of the response of an individual to the therapy. Thus, to analyze or predict the probable amount of growth that can be expected during treatment, researchers have developed prediction models [61]. Ranke et al. developed and validated a GH treatment prediction model for patients born with SGA using the data of children from the KIGS (Pharmacia International Growth Database) and/or those who participated in previous clinical trials. This model inferred that GH dose is the crucial factor for response prediction [62]. Despite possessing several advantages associated with the use of prediction models, their use in clinical practice is still limited. The unavailability of user-friendly software systems and the lack of prediction models for the Indian population deter physicians from considering the use of these prediction models [61].

**Figure 3:**
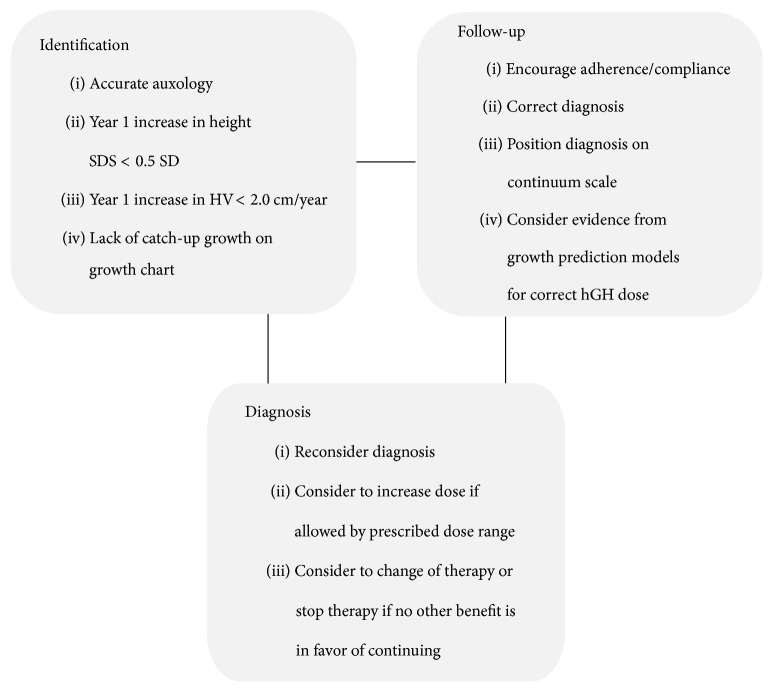
Identification, prevention, and management of poor response to growth hormone therapy. Bang et al. [15]. SDS: standard deviation score; SD: standard deviation; HV: height velocity; hGH: human growth hormone.

The second important challenge which limits the effectiveness of GH therapy is patient adherence. A literature search has found several studies which identified that poor adherence is the major factor that reduces the effectiveness of GH therapy [63, 64]. Aydin et al. conducted a multicentric study on 217 GH-naïve patients to assess adherence to GH therapy. The study found poor adherence to the therapy and determined it to be the underlying factor responsible for suboptimal growth during therapy [63]. A systematic review by Fisher and Acerini also observed that adherence to GH therapy is suboptimal. It could not identify the cause of nonadherence and recommended further research to be conducted [65]. Several factors are known to be associated with nonadherence to the therapy such as type of delivery system, discomfort with injections, cost of treatment, socioeconomic status, lack of communication/training from healthcare providers, poor understanding of disease and consequences of missed doses, requirement for long-term treatment, and lack of immediate clinical improvement and peer or psychosocial pressure (e.g., during adolescence) [57, 65]. Poor response to GH therapy can be identified and managed as presented in Figure 3.

Bozzola et al. suggested that regularly interviewing GHD patients could be a useful approach to improve adherence and also mentioned that communication with patients and their parents should be in a nonaggressive manner [66]. Muller et al. conducted a randomized crossover study comparing a liquid formulation of GH with the older freeze-dried product and found that an overall preference was given to the liquid product, with 98% patients rating it as easier to use [67]. In another study by Iyoda et al., 85% of the patients found liquid preparation to be more convenient for use [68]. Injection pain is one of the major factors that influence compliance. Optimization of the preservative and buffer content of a liquid GH formulation may reduce injection pain and, hence, improve patient compliance [69].

An observational study assessed treatment adherence with the Easypod™ in children and their views regarding its use. A total of 87.5% patients showed adherence to the therapy during the 3-month period of the treatment. More than 80% of children reported it to be easy to use, speedy, and comfortable. This device is unique as it helps in tracking the daily injections of the medication and thus can help in assessing whether it has been taken as prescribed. This device can therefore help to improve adherence to therapy [70].

A study on the use of recombinant somatotropin (r-hGH) as a long-term therapy (11 years) to treat GHD reported positive catch-up growth response and bone age acceleration in accordance with age versus height age [71]. The median HSDS improved significantly from −3.8 at baseline to −3.3 (*p* < 0.001) during the first year of r-hGH therapy and improved further to −1.5 after 7 years of the therapy.

### 3.3. Expert Opinion

This review has summarized the challenges associated with the timely diagnosis of GHD and treatment. Experts at the advisory meetings also presented their suggestions to improve early diagnosis of GHD, optimization of GH therapy, and patient adherence.

#### 3.3.1. Advisory Board Suggestions on Early Diagnosis of GHD

The panel pointed out the lack of public education. Basic education to the public on the awareness of growth monitoring will aid in improving referrals of the children. Suggestions to improve referral were based around early enough awareness of height delay, with one or two critical time points during infancy, in children 4-5 years of age and before the onset of puberty, when early intervention would aim to improve adult height. Several schools have initiated a regular height and weight measurement activity, the data for which are collected and stored in the database owned by CBSE. However, these data are underutilized and not transferred to healthcare institutions. Simple tools such as electronic Excel databases which allow data to be collected and make a simple analysis by selecting subgroups of children with SS (e.g., below 3rd or 5th centile of height for chronological age) will be useful. These tools will improve referral of children with SS to secondary care and eventually improve late diagnosis of disorders associated with SS beyond GHD. Advisors suggested having further collaboration with the Endocrine Society of India (ESI) to implement joint efforts to improve referral of children with SS related disorders. They also opined that screening guidance needs to be revisited and should endorse collaboration with IAP, the ESI, Indian Society for Pediatric and Adolescent Endocrinology (ISPAE), and CBSE electronic health record data.

At advisory board meetings, questions on awareness about the best practice of using stadiometers were also raised (e.g., selection of the right stadiometer, measurement technique, and stadiometers that do not pass quality control). A stadiometer, which consists of a vertical ruler with a sliding horizontal rod, is a tool to measure height accurately [1]. Since accurate measurement of height is the key to diagnose growth disorders in a child and is usually done by nurses and primary care physicians, it is of great importance that these healthcare providers are trained in the minimal standards of good quality stadiometers and height measurement techniques. Plotting of the growth chart helps in assessing a child's growth pattern over time. The advisory boards reinforced the use of Indian charts. Agarwal et al. have developed charts that can help in monitoring the growth from birth up to the age of 18 years, unlike the WHO charts that can be used to monitor growth only up to the age of 5 years [34, 72]. These charts are recognized to be the best charts for growth monitoring in Indian children. The IAP should encourage all its members to carry out growth monitoring regularly. The IAP growth chart committee recommends the use of revised growth charts for height, weight, and body mass index (BMI) for children in the age group of 5–18 years as suggested by Khadilkar et al. and WHO standards for growth assessment in children below 5 years of age [31].

The panel also emphasized the need to create awareness among parents to remain cautious if the child is growing well till the age of 4, suggesting that parents should initiate monitoring if they then observe that the child is not growing well after the age of 4 years. A visit to a specialist is recommended at least 1 year before the start of puberty (i.e., 8 years in girls and 9 years in boys) [34].

It was suggested by the panel members that it should be the social responsibility and joint efforts of all stakeholders (government, schools, and healthcare institutions) to implement an effective screening system that includes height as one of the measurements. This screening should include measurements of height in addition to weight, eye sight, and dental care. The use of DCG criteria for referral suggested that a large percentage of the referrals were mainly due to the deflection of length during the first 3 years of life [38]. Nevertheless, availability of growth charts, awareness, and training of medical personnel to measure growth properly and plotting data on the growth chart was agreed to be the cornerstone of success towards early diagnosis/detection of growth disorders [31, 34].

Although several diagnostic tests are available for the diagnosis of GHD, none of them can be relied upon independently. In one of the advisory board meetings it was suggested that diagnosis of GHD should be based on multiple diagnostic criteria, since there is no standard diagnostic test. Experts also mentioned the need to improve availability and standardization of GH, IGF-1, and IGFBP-3 assays for the diagnosis of GHD. GH provocation testing lacks significance since the tests are reported to be poorly reproducible. Use of different stimuli results in variability in the results of different GH assays. In addition, the results are also affected by the pattern of GH secretion prior to the administration of the stimulus. Short-term nutrition is another important factor that affects the plasma concentration of IGF-1, which is reduced by undernutrition even with high GH secretion. Thus, there is a need for other tests to accurately assess GHD [45, 73]. A review by Clemmons reported obstacles in GH and IGF-1 standardization which include use of different calibrator materials, varying results with the assays because of the different antibody types used that bind to different forms of GH and IGF-1 and the effect of matrix component. Binding proteins including GH-binding protein (GHBP) and IGFBPs also interfere with the assay findings. Thus, to improve the standardization of the different assays, these obstacles need to be overcome [74].

#### 3.3.2. Advisory Board Suggestions on Improving Treatment Adherence

Patient compliance is of critical importance to ensure benefits of the treatment. In the past, limited availability of GH was a barrier to the optimal therapy of GHD. Today, we have entered an era of virtually unlimited supply of GH although the cost still remains a limiting factor. The advisory board suggested the following:

- A therapy device that is easy to use and appealing to children would greatly help in improving patient adherence
- Reduce the cost of GH therapy and support the use of GH treatment by the government
- Designing robust patient support programs will help to achieve good adherence
- Organize educational activities (educational camps) for patients and parents
- Social media can be a good mode to spread awareness about the early diagnosis and treatment of GHD
- Patients should be asked to use calendars to improve adherence to the therapy and doctors can take the initiative to send reminders to the patients* via* text messages on their phones for their upcoming appointments
- Increased family and social support to the patient can also help in improving adherence

## 4. Conclusion

To date no single test has been developed that can be considered to be definitive in diagnosing GHD. Therefore, more research needs to be conducted to develop a robust diagnostic criterion for GHD. Further, there is a need for database and registries for monitoring various possible ethnicity-specific growth responses and adverse effects (AEs). India-specific databases or registries may help provide epidemiological data for GHD in an Indian context.

Early initiation of therapy could better the chances of achieving final adult height. Education and awareness about growth disorders among parents would help improve the diagnosis and treatment of children with GHD. The first point of contact in a patient's journey is that physician/pediatrician needs to be well equipped to identify cases of GHD. Encouraging the use of prediction models by pediatricians/endocrinologists will help in optimizing the treatment. Physicians in India should be given regular training and more emphasis should be put on using the Indian growth charts for growth monitoring. Further, compliance to treatment is one of the major obstacles in poor growth response. The availability of an easy-to-use delivery system would be beneficial in improving adherence and achieving satisfactory or optimal outcomes.

## Figures and Tables

**Table 1:** Various tests for diagnosis of growth hormone deficiency.

Study	GH assay	IGF-1 assay	Stimulation tests	Number of patients	Mean age (yrs)
Raghupathy [23]	—	—	LHRH (luteinizing hormone–releasing hormone), TRH (thyrotropin-releasing factor)	8	13.8
Kota et al. [24]	Solid-phase, 2-site CIA	Solid-phase, enzyme-labeled CIA	IIH, clonidine	25	8.6 ± 2.9 years
Ekbote et al. [25]	Solid-phase, 2-site CIA	Solid-phase, enzyme-labeled CIA	Clonidine, glucagon	28	8.6
Khadilkar et al. [26]	—	—	Stimulation tests (type not mentioned) or one test with typical phenotype	15	12
Menon et al. [27]	—	RIA	IIH, clonidine	20	9.43 ± 3.52 years
Bajpai et al. [28]	—	—	IIH, clonidine	96	9.9 ± 3.7 years
Garg et al. [29]	—	—	IIH, clonidine/exercise	71	10.07 ± 3.26 years
Kannan et al. [30]	RIA	—	IIH, clonidine, diazepam	30	2–14

CIA: chemiluminescent immunometric assay; IIH: insulin induced hypoglycaemia; RIA: radioimmunoassay.

**Table 2:** Advantages and disadvantages of Indian growth reference charts.

Advantages	Disadvantages
Growth reference monitoring data is helpful in diagnosing overweight, undernutrition, obesity as per existing growth pattern of the children India growth charts are contemporary and are true presentation of current growth pattern Cut-offs for body mass index (BMI) have been kept lowered by Indian charts (23 Kg/m^2^ and 27 Kg/m^2^) as suggested by the World Health Organization (25 Kg/m^2^ and 30 Kg/m^2^) —	All children grow at different pace; variations in growth occur among children due to difference in ethnicity, nutrition intake, and environment. Thus, there are chances that some children may remain undiagnosed With time, the children growth pattern changes. Thus, the reference data needs to be updated regularly The limitation of the Indian Academy of Pediatrics (IAP) revised growth chart for the age group 5–18 yr is that the study designs and measurement scales in the nine studies included are different. However, methodology of these studies was carefully assessed to reduce the risk of errors while performing data analysis The growth reference for children under the age 5 adopted by the IAP was developed based on the data collected between years 1989–1991. As the reference data needs to be updated regularly, the use of these growth references is not recommended now

V. Khadilkar et al. [31–33].
